# Activity of a Medical Relief Team from Shizuoka Hospital that was Dispatched to the Noto Peninsula Earthquake in Reiwa 6 (2024)

**DOI:** 10.14789/jmj.JMJ24-0006-OT

**Published:** 2024-05-10

**Authors:** YOUICHI YANAGAWA, IKUTO TAKEUCHI, YOSHIKI NAGASAWA, KAZUKI HORII, MOTOHIRO ISHIBASHI, TAIKI ASAKA, SHINYA TADA, MISAO SAKURAI, HARUMI KATO, YOKO NOZAWA, AKIO KANDA, HIROMICHI OHSAKA

**Affiliations:** 1 Department of Shizuoka Hospital, Juntendo University, Shizuoka, Japan; 1 Department of Shizuoka Hospital, Juntendo University, Shizuoka, Japan

**Keywords:** medical relief team, Noto, earthquake

## Abstract

**Objective:**

The present study aimed to report on the activity of a medical relief team from Juntendo Shizuoka Hospital that was dispatched to the Noto Peninsula Earthquake in Reiwa 6.

**Design:**

Narrative report.

**Results:**

The activities conducted on-site in the Noto Peninsula involved multiple deployments of the Juntendo University Shizuoka Hospital Disaster Medical Assistance Team (JS-DMAT). The first deployment from January 2nd to January 6th faced challenges due to damaged infrastructure, particularly roads, affecting mobility. The team focused on hospital medical support, patient transportation, and DMAT headquarters assistance. The second deployment, from January 8th to January 12th, encountered persistently damaged roads, leading to incidents but no significant vehicle damage. The team engaged in screening, zoning, medical examinations, and DMAT headquarters support in evacuation shelters. The third team's planned activities in early February were canceled by Shizuoka Prefecture.

Additionally, on January 7, 2024, personnel from Juntendo Shizuoka Hospital participated in the Shizuoka Prefectural DMAT Coordination Headquarters activity, documenting DMAT activities and assessing team members' health. The Ministry of Health, Labour and Welfare's request for the fourth Shizuoka Prefecture DMAT dispatch led to the selection of the second JS-DMAT for deployment.

**Conclusion:**

The activities related to the Noto Peninsula earthquake by JS-DMAT were reported. Lessons from this disaster are being sought to guide future disaster response preparations.

## Introduction

On January 1, Reiwa 6, at 16:10, the Noto Peninsula Earthquake with an estimated magnitude of 7.6 occurred. This seismic event resulted in an expansion of approximately 4.4 square kilometers of land towards the sea within a range of about 90 kilometers on the northern side of the Noto Peninsula^1)^. The maximum coseismic displacement was observed in Wajima City, Ishikawa Prefecture, measuring approximately 240 meters. Furthermore, the ground uplift in Wajima City reached a maximum of 3.9 meters, marking the most significant uplift in the past 6,000 years^1)^. The earthquake was characterized by a seismic intensity of 7 in Shika Town, Ishikawa Prefecture, and coastal areas experienced tsunamis, causing widespread damage. As of February 28, 2024, it has been confirmed that there were more than 241 fatalities, over 1,540 injuries, and damage to over 77,703 residences due to this seismic event.

The Ministry of Health, Labour and Welfare requested the dispatch of Disaster Medical Assistance Teams (DMAT) to the central region on January 2, 2024, including Shizuoka Prefecture, in response to the Noto Peninsula Earthquake. The DMATs are mobile, trained medical teams that can be rapidly deployed during the acute phase of a sudden-onset disaster^2)^. The Shizuoka prefecture government requested the dispatch of Shizuoka DMATs. The Juntendo Shizuoka DMAT (JS- DMAT) responded to a request from the Shizuoka prefecture government. We hereby present the actions taken by JS-DMAT in response to the Noto Peninsula Earthquake in Reiwa 6.

## Report

Shizuoka University Hospital, affiliated with Juntendo University School of Medicine, carried out DMAT related activities in response to the Noto Peninsula Earthquake, commissioned by the Shizuoka Prefectural Government. These activities can be broadly classified into three main categories. Firstly, there were those who were dispatched to the Noto Peninsula earthquake-affected area and engaged in on-site operations. Secondly, there were activities coordinated at the Shizuoka Prefectural DMAT Coordination Headquarters. The last category involved medical helicopter operations in the eastern part of Shizuoka Prefecture.

### (1) Activities conducted on-site in the Noto Peninsula

#### a. The First Juntendo University School of Medicine Affiliated Shizuoka Hospital DMAT Activity (Figure 1)

The first dispatch of the Juntendo University School of Medicine Affiliated Shizuoka Hospital DMAT (Disaster Medical Assistance Team) took place from January 2nd to January 6th. The chronological details of this activity are summarized in Table 1. The operation faced challenges due to significant damage to key infrastructure, particularly roads and sewage systems, among the lifelines in the disaster-stricken area. The notable deterioration of roads posed substantial difficulties for mobility, consuming considerable time. The team primarily engaged in hospital medical support (Figure 2), patient transportation, and support for the DMAT headquarters in the affected area.

**Figure 1:**
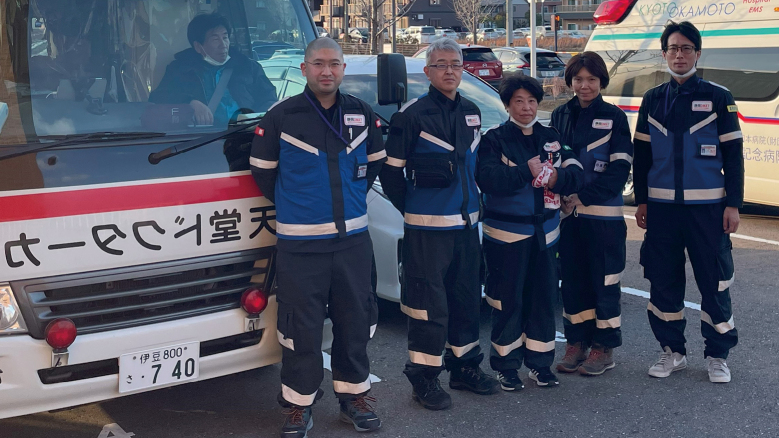
The First Juntendo University School of Medicine Affiliated Shizuoka Hospital DMAT (JS-DMAT) The first JS-DMAT was consisted of one physician, two nurses, and two logistics personnel. DMAT, Disaster Medical Assistance Team

**Table 1:** Activities of the First Juntendo Shizuoka DMAT

Dates and times	Contents of activities
2024/1/2 19:27	The Juntendo DMAT team departed with one DMAT vehicle and one ambulance.
2024/1/3 0:10	They stayed overnight in Gunjo City, Gifu Prefecture.
2024/1/3 5:58	Upon leaving the accommodation, they encountered a road closure for general vehicles beyond the Takaoka interchange on the Noto Expressway. The team manually moved cones to pass through.
2024/1/3 9:20	Upon arrival at Noto General Hospital (Headquarters for Noto Medical Care Area Activities), they found a chaotic situation with confusion in information and inadequate coordination with administrative and health authorities. The directive for deployment to the arriving DMAT could not be executed. While electricity was available, there was a continuous water outage. The toilet conditions were severe, with overflowing waste, prompting the team to use standby time to initiate improvement activities.
2024/1/3 12:58	They began investigating the damage situation in the Noto medical care area and updating the report form.
2024/1/3 14:10	Patient transport for a femoral neck fracture was requested to Ishikawa Prefectural Central Hospital. Some team members remained at the headquarters to continue supporting operational tasks.
2024/1/3 15:38	Patient transport commenced.
2024/1/3 16:39	They originally planned for Ishikawa Central Hospital. However, the destination was changed to Kanazawa University Hospital, leading to a change in the route.
2024/1/3 16:56	Upon arrival at Kanazawa University Hospital, the team faced a shortage of gasoline, resulting in a few hours spent searching for a gas station in Kanazawa.
2024/1/4 6:02	The team members who conducted transports in the same area stayed overnight. They then departed for Noto General Hospital.
2024/1/4 6:53	They arrived at their destination.
2024/1/4 9:01	The dispatch of four teams from Juntendo Shizuoka Hospital DMAT, led by the leader, was decided. The teams were set to go to Wajima General Hospital via Anamizu General Hospital, with Gifu Prefectural General Medical Center, Yamanashi Prefectural Central Hospital, and Shizuoka Red Cross DMAT. During this dispatch, it was also decided that the transportation of insufficient supplies and food would be carried out in addition.
2024/1/4 10:02	Departing from Noto General Hospital, the roads to Anamizu were frequently blocked, requiring assistance from the Ministry of Land, Infrastructure, Transport and Tourism, the Self-Defense Forces, and the police. The team proceeded towards their destination with the help of these forces.
2024/1/4 13:27	After arriving at Anamizu General Hospital and leaving supplies, the team departed for Wajima General Hospital. The journey to Wajima General Hospital was challenging, with severe road damage, collapsed houses, and landslides blocking the way. The team encountered life-threatening situations due to falling rocks and debris from collapsed houses. There were numerous areas with the risk of tire bursts, and they had to traverse cracked roads multiple times. The normally 2-hour journey took over 7 hours, and they reached their destination in the evening. The road they traveled was the only passage, repeatedly affected by road collapses and landslides. This mission posed the risk of being unable to return in case of road closures due to aftershocks or rainfall. The situation required meticulous attention to avoid tire punctures.
2024/1/4 15:45	Arrived at Wajima General Hospital. The hospital did not experience a power outage, and though there was a water cut, water supply was still available. The major issue at the hospital was related to toilets due to problems with the sewage system. While the hospital managed to provide emergency care, continuing hospitalization was challenging. As a result, patients eligible for hospitalization were being transferred to other medical facilities as needed. At Wajima Hospital, staff who had been on duty on New Year’s Day continued working until the 4th. Despite their homes being damaged, the staff continued their duties without returning home. The surrounding evacuation centers were also in poor conditions, and the situation was challenging for both the city and the health department. Confirming the safety of residents in collapsed houses was almost impossible at that time.
2024/1/4 16:30	They attended a meeting, and as a result, it was decided that they would be responsible for the overnight emergency room while taking breaks for rest. They were able to conduct CT scans using a self-power generator, but the images couldn’t be confirmed on the electronic medical record. Therefore, they performed the image interpretation in the CT examination room. They handled cases such as suturing, treating burns from prolonged application of hot packs in evacuation centers, and managing a clavicle fracture resulting from a fall in a precarious location. Due to the unavailability of the electronic medical record system, I documented alternative information to referral letters.
2024/1/5 8:00	They participated in a meeting and received a request for the transfer of a patient with diabetic ketoacidosis. To ensure space for patient transport, they donated essential items from the carried equipment that were needed in the disaster-stricken area. When a request for accommodating a patient with diarrhea came in, and considering the unavailability of functional toilets, they provided a portable, seated toilet brought from our hospital to meet the specific needs of the situation.
2024/1/5 11:22	Patient transport to Ishikawa Prefectural Central Hospital was initiated.
2024/1/5 15:31	Arrival at Ishikawa Prefectural Central Hospital.
2024/1/5 16:10	After completing the patient transport, the conclusion of the transfer was reported to Noto General Hospital. The team received instructions to withdraw from the headquarters.
2024/1/5 18:28	Stayed overnight in Himi City.
2024/1/6 7:30	Departure from the accommodation.
2024/1/6 15:15	Returned to Juntendo Shizuoka hospital.

DMAT, Disaster Medical Assistance Team

**Figure 2:**
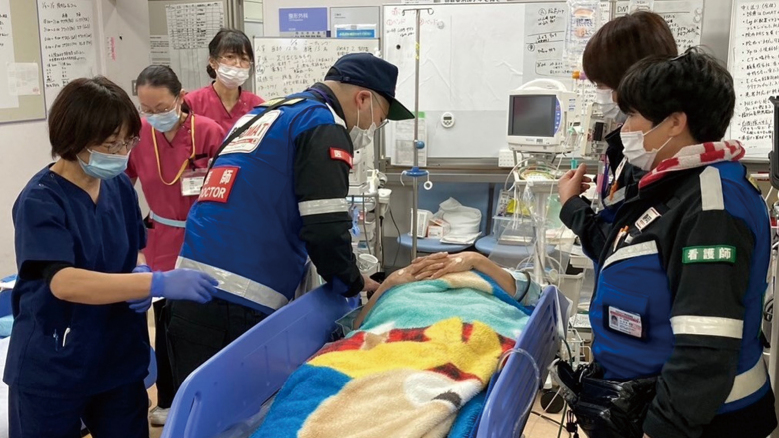
The First Juntendo University School of Medicine Affiliated Shizuoka Hospital DMAT Activity The photo depicts a scene of medical services being conducted at a disaster response hospital. DMAT, Disaster Medical Assistance Team

#### b. The Second Juntendo University School of Medicine Affiliated Shizuoka Hospital DMAT Activity (Figure 3)

The second deployment of the Juntendo University School of Medicine Affiliated Shizuoka Hospital DMAT took place from January 8th to January 12th. The chronological details of this activity are outlined in Table 2. Similar to the first deployment, the operation faced challenges due to persistently damaged roads in the disaster-stricken area. There were incidents of wheel dislocation during nighttime travel, attributed to encountering rockfall and difficult snow-covered roads. Fortunately, the vehicle sustained no significant damage. The team primarily engaged in activities such as screening (Figure 4), zoning, medical examinations, and support for the DMAT headquarters in evacuation shelters in the affected region.

**Figure 3:**
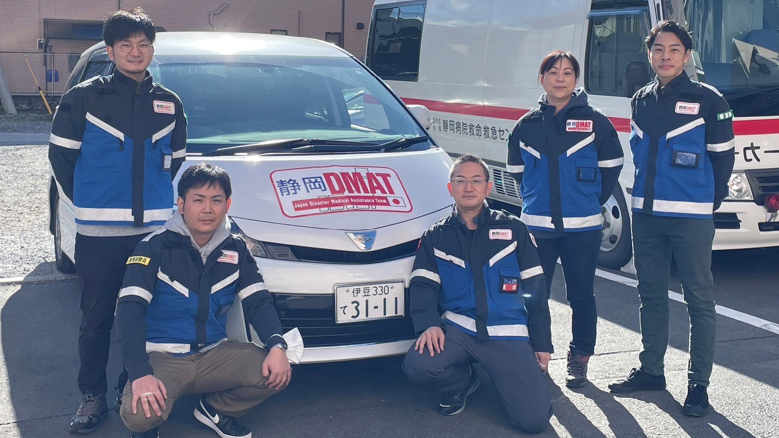
The Second Juntendo University School of Medicine Affiliated Shizuoka Hospital DMAT (JS-DMAT) The second JS-DMAT was consisted of one physician, three nurses, and one logistic personnel. DMAT, Disaster Medical Assistance Team

**Table 2:** Activities of the Second Juntendo Shizuoka DMAT

Dates and times	Contents of activities
2024/1/8 9:05	Received the fourth deployment order and began preparations.
2024/1/8 12:10	Departed from Juntendo University School of Medicine, Shizuoka Hospital.
2024/1/8 21:30	Arrived at the accommodation in Takaoka.
2024/1/9 5:55	Departed from the accommodation.
2024/1/9 7:33	Arrived at Noto General Hospital, the base of operations.
2024/1/9 8:30	Departed from Noto General Hospital towards the Wajima City Hall, combining material transport.
2024/1/9 12:03	Arrived at the Health, Medical, and Welfare Coordination Headquarters within Wajima City Hall.
2024/1/9 13:50	Initiated activities by dividing into two groups for shelter support and assistance to the headquarters. Conducted medical examinations for 8 individuals with health issues at Kawai Elementary School. At Najimi Evacuation Center (Community Center), performed screening for 400 individuals (2 with fever, 2 with diarrhea, and 1 requiring treatment for a hand injury) and provided medical examinations.
2024/1/9 21:19	Concluded medical examinations and screening. Started moving towards the accommodation. On the way back, the team encountered challenges such as the vehicle riding over fallen rocks and a wheel detaching, but the team worked together to resolve the issues.
2024/1/10 0:36	Arrived at the accommodation in Wajima City.
2024/1/10 7:56	Departed from the accommodation.
2024/1/10 8:11	Received requests for screening at evacuation centers.
2024/1/10 8:51	Split into two teams and departed from Wajima City Hall towards Mitsui Community Center and Noto Airport Aid Station.
2024/1/10 10:38	Arrived at Noto Airport Aid Station and began screening for 30 evacuees.
2024/1/10 11:43	Concluded screening at Noto Airport Aid Station and departed for Mitsui Community Center.
2024/1/10 13:28	Arrived at Mitsui Community Center. Started screening for 80 evacuees and conducted examinations for those with symptoms such as fever and vomiting.
2024/1/10 15:10	Concluded activities at Mitsui Community Center Evacuation Center.
2024/1/10 16:00	Arrived at Wajima City Hall.
2024/1/10 16:35	Departed from Wajima City Hall towards Houshi Elementary School Evacuation Center.
2024/1/10 16:48	Arrived at Houshi Elementary School Evacuation Center with 211 evacuees. Due to the increasing trend of respiratory infections, zoning was implemented. There were 9 positive cases for influenza, 12 positive cases for COVID-19, and 1 case positive for both.
2024/1/10 21:50	Concluded activities and departed for Wajima City Hall.
2024/1/10 22:05	Arrived at Wajima City Hall.
2024/1/10 22:20	Departed from Wajima City Hall to the accommodation in Wajima City.
2024/1/10 22:42	Arrived at the accommodation.
2024/1/11 7:42	Departed from the accommodation.
2024/1/11 7:53	Arrived at the Operational Base within Wajima City Hall.
2024/1/11 9:53	Split into two groups; one went to Mitsui Community Center for zoning, and the other to Monzen Community Center for screening. Departed from Wajima City Hall.
2024/1/11 11:18	Arrived at Mitsui Community Center. Two individuals with fever and several with cold symptoms were identified. Those with fever were isolated and moved to other facilities. Health nurses and infection control nurses were dispatched to the facility for information sharing. Completed zoning and moved to Monzen Community Center.
2024/1/11 12:20	Arrived at Monzen Community Center, joined with the other group, and conducted screening at evacuation centers in the Monzen area. Two individuals with fever among evacuees at Monzen Community Center requested examination. The results confirmed both were positive for influenza. Conducted screening at Fukada Assembly Hall and Nishinakao Evacuation Center in the Monzen district.
2024/1/11 16:13	After completing screening at the planned locations, departed for the Health, Medical, and Welfare Coordination Headquarters.
2024/1/11 17:23	Arrived at Wajima City Hall.
2024/1/11 18:30	Attended a regular meeting.
2024/1/11 19:48	Concluded today’s activities. This deployment is now completed.
2024/1/11 22:50	Arrived at the accommodation in Takaoka.
2024/1/12 9:00	During this mission, there were incidents involving wheel detachment and rockfall, so an automobile company in the city were inspected.
2024/1/12 10:15	Departed for Juntendo Shizuoka Hospital.
2024/1/12 17:31	Returned to Juntendo Shizuoka hospital.

DMAT, Disaster Medical Assistance Team

**Figure 4:**
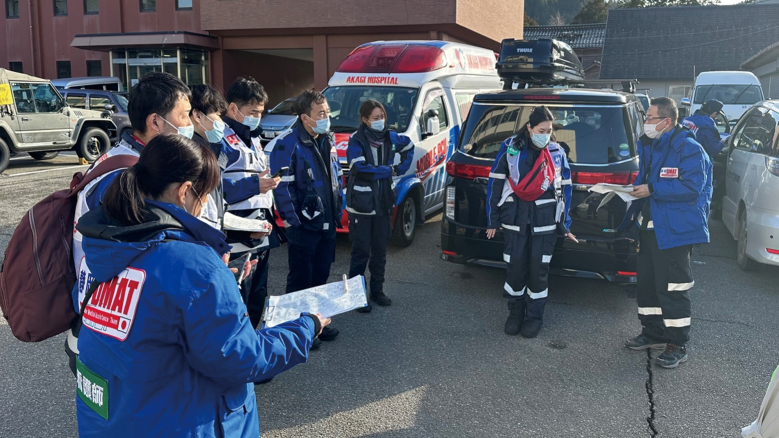
The Second Juntendo University School of Medicine Affiliated Shizuoka Hospital DMAT Activity The photo captures a scene of a meeting discussing screening procedures at an evacuation center. DMAT, Disaster Medical Assistance Team

#### c. The Third Juntendo University School of Medicine Affiliated Shizuoka Hospital DMAT Activity

The third team, initially scheduled to commence operations in early February, received notice from Shizuoka Prefecture on January 23rd that the DMAT dispatch would be canceled because the Ministry of Health, Labour and Welfare was unable to forecast the demand for support by DMAT in the Noto Peninsula earthquake. As a result, the planned activities for the third team were canceled.

### (2) The Shizuoka Prefectural DMAT Coordination Headquarters activity (Figure 5, Table 3)

On January 7, 2024 (Saturday), as per the request from Shizuoka Prefecture, medical and logistics personnel in Juntendo Shizuoka Hospital participated in the Shizuoka Prefectural DMAT Coordination Headquarters activity. The objectives of this activity included documenting and understanding the activities of the DMAT dispatched from Shizuoka Prefecture and assessing the health status of the team members.

**Figure 5:**
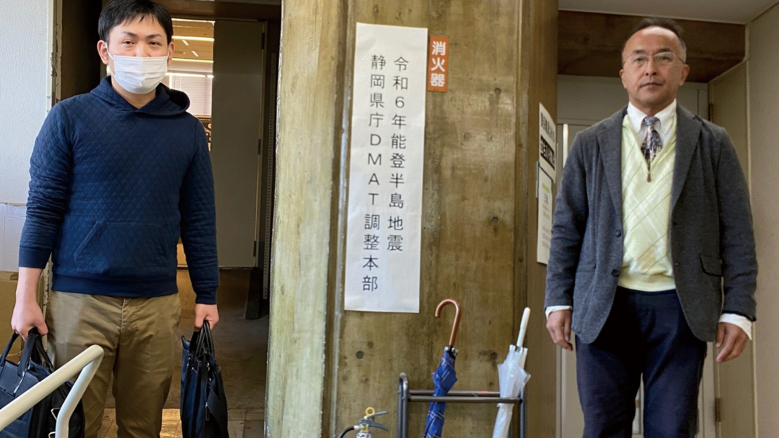
Scene at the Shizuoka Prefectural DMAT Coordination Headquarters One doctor and one logistic personnel One physician from Juntendo University School of Medicine Affiliated Shizuoka Hospital’s DMAT (JS-DMAT), serving as the head of the team, along with three logistics personnel (including one from the JS-DMAT) participated in support operations for several Shizuoka DMATs, dispatched to the Noto Peninsula Earthquake. This took place at the Shizuoka DMAT Coordination Headquarters, where they collaborated with local government officials. DMAT, Disaster Medical Assistance Team

**Table 3:** The activity of Shizuoka Prefecture DMAT Coordination Headquarters

Dates and times	Contents of activities
2024/1/7 8:40	Arrival at Shizuoka Prefectural Government Office
2024/1/7 8:45	Arrival at the Shizuoka Prefectural Government
2024/1/7 9:00	DMAT Coordination Headquarters Morning routine meeting at the Shizuoka Prefectural Government
2024/1/7 9:10	DMAT Coordination Headquarters Information gathering regarding the DMAT activities dispatched by Shizuoka Prefecture for the Noto Peninsula earthquake
2024/1/7 12:38	Shizuoka Prefecture received a dispatch request for the third team of DMAT from the Ministry of Health, Labour and Welfare, and coordinated the dispatched DMAT.
2024/1/7 17:45	Evening routine meeting at the Shizuoka Prefectural Government DMAT Coordination Headquarters
2024/1/7 19:30	Withdrawal

DMAT, Disaster Medical Assistance Team

On the same day, the Ministry of Health, Labour and Welfare issued a request for the fourth dispatch of Shizuoka Prefecture DMAT. In response, contacts were made with disaster base hospitals within Shizuoka Prefecture to determine and finalize the DMAT teams to be dispatched. The team from Juntendo University School of Medicine Affiliated Shizuoka Hospital DMAT, which was the second dispatch team, was among those selected for deployment.

### (3) The Shizuoka Prefecture Eastern Doctor Helicopter

On January 3, 2024, Ishikawa Prefecture made a support request to Shizuoka Prefecture based on the basic agreement regarding wide-area collaboration in the event of a large-scale disaster, specifically related to doctor helicopter assistance. Shizuoka Prefecture is equipped with two doctor helicopters, with the Shizuoka Prefecture Eastern Doctor Helicopter using our hospital as its base facility. The Shizuoka Prefecture Eastern Doctor Helicopter ranks second nationally in terms of patient transportation among the 56 doctor helicopter bases, consistently having lower deployment numbers due to the high patient transport volume. On the other hand, the number of dispatches for the Shizuoka Prefecture Western Doctor Helicopter is approximate one-fifth of that in the eastern part, ranking it among the lowest in the area among Doctor Helicopter bases. This is because there are five functioning emergency medical centers in western Shizuoka Prefecture, and the transportation of emergency patients by land is sufficient. In contrast, in the eastern part, there is only one functioning emergency medical center at our hospital, and the frequent use of Doctor Helicopters for transporting emergency patients from distant locations is reflected.

In response to the request, the Shizuoka Prefecture Western Doctor Helicopter, which is also geographically closer to Ishikawa Prefecture, was dispatched to provide support. To cover the vacuum created by the Western Doctor Helicopter's deployment, the Eastern Doctor Helicopter extended its coverage to include the western region. Notably, during the period when the Western Doctor Helicopter was assisting in Ishikawa Prefecture, there were no requests for doctor helicopter support from the western region of Shizuoka Prefecture.

## Discussion

JS-DMAT's operational challenges approximately 1.5 days after the disaster onset included difficulties in local access due to disrupted lifelines, particularly the challenges of vehicular movement caused by road damage, problems in waste disposal due to sewage system destruction, and communication disruptions. In the midst of these extensive damages, providing appropriate medical care requires a comprehensive approach that goes beyond medical specialization. Dealing with such situations necessitates not only medical preparedness but also coordinated efforts involving various organizations^3)^. Indeed, what is crucial for acute-phase disaster healthcare is not just medicine itself, but rather collaborative efforts through integrated organizational structures^4)^.

The primary issue arose from the difficulty in collecting information from evacuation centers due to communication breakdowns. Additionally, direct on-site information gathering was hindered by road damage, especially in areas where the extent of road damage was severe, making information collection impossible. While aerial approaches are an option when land routes are challenging for information gathering, the Noto region, where the disaster occurred, faced obstacles due to low- hanging clouds and adverse weather conditions, exacerbated by the short daylight hours and heavy snowfall in January^5)^.

On the medical front, the focus shifted from the peak period of acute trauma response in the ultra-acute phase to addressing acute illnesses, including infectious diseases among evacuees living in shelters, and understanding the overall health status, similar to a previous report^6)^. Trauma cases from falling objects and collapsed houses primarily peaked within the first 24 hours of the ultra-acute phase. Since JS-DMAT arrived on-site 1.5 days after the disaster, there was no significant need for trauma care.

The Noto Peninsula, already a sparsely populated area with an aging population, now faces significant disruption to lifelines due to a seismic event, especially with an expected prolonged restoration period for water and sewage systems. Consequently, secondary evacuations to unaffected areas are underway due to numerous individuals unable to return home from evacuation centers. The Izu Peninsula, where JS-DMAT is based and shares similar topography with the Noto Peninsula, is using this disaster as a lesson to explore future disaster response preparations^7)^.

Regarding the dispatch of personnel from our hospital during the acute phase of the Noto Peninsula earthquake, the dispatched staff sacrificed their New Year holidays and vacations to engage in DMAT activities. The impact of scheduling adjustments affected not only the dispatched personnel but also the staff remaining at the hospital, but they supported relief efforts indirectly by helping each other. Our hospital is the last stronghold of emergency medical care in eastern Shizuoka Prefecture, and during this disaster dispatch period, we continued to accept emergency cases as usual, with an ambulance refusal rate of less than 1%, which is normal. For this dispatch, the personnel costs, fuel expenses, and allowances for medications used by the dispatched staff are covered under the Disaster Relief Act.

## Conclusion

The activities related to the Noto Peninsula earthquake by JS-DMAT were reported. Lessons from this disaster are being sought to guide future disaster response preparations.

## Funding

This work was supported in part by a Grant-in- Aid for Special Research in Subsidies for ordinary expenses of private schools from the Promotion and Mutual Aid Corporation for Private Schools of Japan.

## Author contributions

IT, YN, KH, MI, DA, ST, MS, HK, YN AK and HO were supervised the work, and YY was a major contributor in writing the manuscript. All authors read and approved the final manuscript.

## Conflicts of interest statement

The authors declare that there are no conflicts of interest.
